# Leucine 434 is essential for docosahexaenoic acid–induced augmentation of L-glutamate transporter current

**DOI:** 10.1016/j.jbc.2022.102793

**Published:** 2022-12-09

**Authors:** Kanako Takahashi, Luying Chen, Misa Sayama, Mian Wu, Mariko Kato Hayashi, Tomohiko Irie, Tomohiko Ohwada, Kaoru Sato

**Affiliations:** 1 Laboratory of Neuropharmacology, Division of Pharmacology, National Institute of Health Sciences, Kawasaki, Kanagawa, Japan; 2 Laboratory of Organic and Medicinal Chemistry, Graduate School of Pharmaceutical Sciences, The University of Tokyo, Tokyo, Japan; 3 Department of Food Science and Nutrition, Faculty of Food and Health Sciences, Showa Women’s University, Tokyo, Japan

**Keywords:** astrocyte, polyunsaturated fatty acid (PUFA), glutamate, transporter, electrophysiology, ALA, α-linolenic acid, ARA, arachidonic acid, DHA, docosahexaenoic acid, DHA-CoA, DHA-Coenzyme A, DHA-EA, DHA-ethanolamide, DHA-Me, DHA methyl ester, DHK, dihydrokainic acid, DPA, docosapentaenoic acid, EAAT2, astrocytic excitatory amino acid transporters 2, EPA, eicosapentaenoic acid, IFD, Induced Fit Docking, IFS, inward facing state, Indo, indomethacin, L-Glu, L-glutamate, OFS, intermediate outward facing state, PDB, protein data bank, PUFA, polyunsaturated fatty acid, TM7b-HP2a, transmembrane regions 7b - re-entrant hairpin loops 2a

## Abstract

Astrocytic excitatory amino acid transporter 2 (EAAT2) plays a major role in removing the excitatory neurotransmitter L-glutamate (L-Glu) from synaptic clefts in the forebrain to prevent excitotoxicity. Polyunsaturated fatty acids such as docosahexaenoic acid (DHA, 22:6 n-3) enhance synaptic transmission, and their target molecules include EAATs. Here, we aimed to investigate the effect of DHA on EAAT2 and identify the key amino acid for DHA/EAAT2 interaction by electrophysiological recording of L-Glu–induced current in *Xenopus* oocytes transfected with EAATs, their chimeras, and single mutants. DHA transiently increased the amplitude of EAAT2 but tended to decrease that of excitatory amino acid transporter subtype 1 (EAAT1), another astrocytic EAAT. Single mutation of leucine (Leu) 434 to alanine (Ala) completely suppressed the augmentation by DHA, while mutation of EAAT1 Ala 435 (corresponding to EAAT2 Leu434) to Leu changed the effect from suppression to augmentation. Other polyunsaturated fatty acids (docosapentaenoic acid, eicosapentaenoic acid, arachidonic acid, and α-linolenic acid) similarly augmented the EAAT2 current and suppressed the EAAT1 current. Finally, our docking analysis suggested the most stable docking site is the lipid crevice of EAAT2, in close proximity to the L-Glu and sodium binding sites, suggesting that the DHA/Leu434 interaction might affect the elevator-like slide and/or the shapes of the other binding sites. Collectively, our results highlight a key molecular detail in the DHA-induced regulation of synaptic transmission involving EAATs.

Docosahexaenoic acid (DHA, 22:6 n-3) is a polyunsaturated fatty acid (PUFA) that is reported to enhance cognition (1, 2), memory (3) and synaptic transmission (4, 5). These studies suggest DHA and PUFAs have multiple target molecules. Among the most-studied molecules are L-glutamate (L-Glu) transporters in the central nervous system (excitatory amino acid transporter subtype 1 [EAAT1]-5 in human) (6, 7, 8). EAATs are a family of excitatory neurotransmitter transporters that take up L-aspartate (L-Asp), D-Asp, and L-Glu to enable convergence of synaptic transmission and to prevent excitotoxicity. In an electrophysiological study using *Xenopus* oocytes, Zarangue *et al.*(9) found that arachidonic acid (ARA) suppresses EAAT1 function and enhances EAAT2 function. In contrast, both ARA and DHA enhance EAAT4 currents (10). In a study of D-[^3^H]Asp uptake by HEK cells transfected with EAAT1, EAAT2, and EAAT3, DHA enhanced the uptake activities of EAAT2 and EAAT3 but inhibited the activity of EAAT1 (11). Among EAATs, EAAT1 and EAAT2 are astrocytic, and EAAT2 mediates L-Glu uptake in the forebrain (12, 13), accounting for ∼90 % of L-Glu uptake in the central nervous system (14, 15). However, it is difficult to maintain stable expression of EAAT2 in cultured cells, so in order to examine the effects of PUFAs such as DHA on EAAT2, as well as EAAT1, we chose to employ electrophysiological studies in *Xenopus* oocytes overexpressing these transporters. The net L-Glu–induced EAAT current is composed of the coupled L-Glu transport current and the uncoupled Cl^−^ (anion) current (16). The coupled L-Glu current is induced by co-transport of 3 Na^+^ and 1 H^+^, followed by the counter-transport of 1 K^+^ (13, 17). The amplitudes of the L-Glu–induced currents of EAAT1 and EAAT2 are linearly correlated with L-Glu transport functions (18). In this study, we aimed to investigate the effect of DHA on EAAT2 and to identify the key amino acid for the interaction between DHA and EAAT2 by means of electrophysiological recording of the L-Glu–induced current in *Xenopus* oocytes transfected with EAATs, their chimeras, and single mutants. We found that Leu434 of EAAT2 has a critical role in the augmenting effect of DHA and the related PUFAs on L-Glu transport.

## Results

### Characterization of the effect of DHA on EAAT2 current

**Figure 1:**
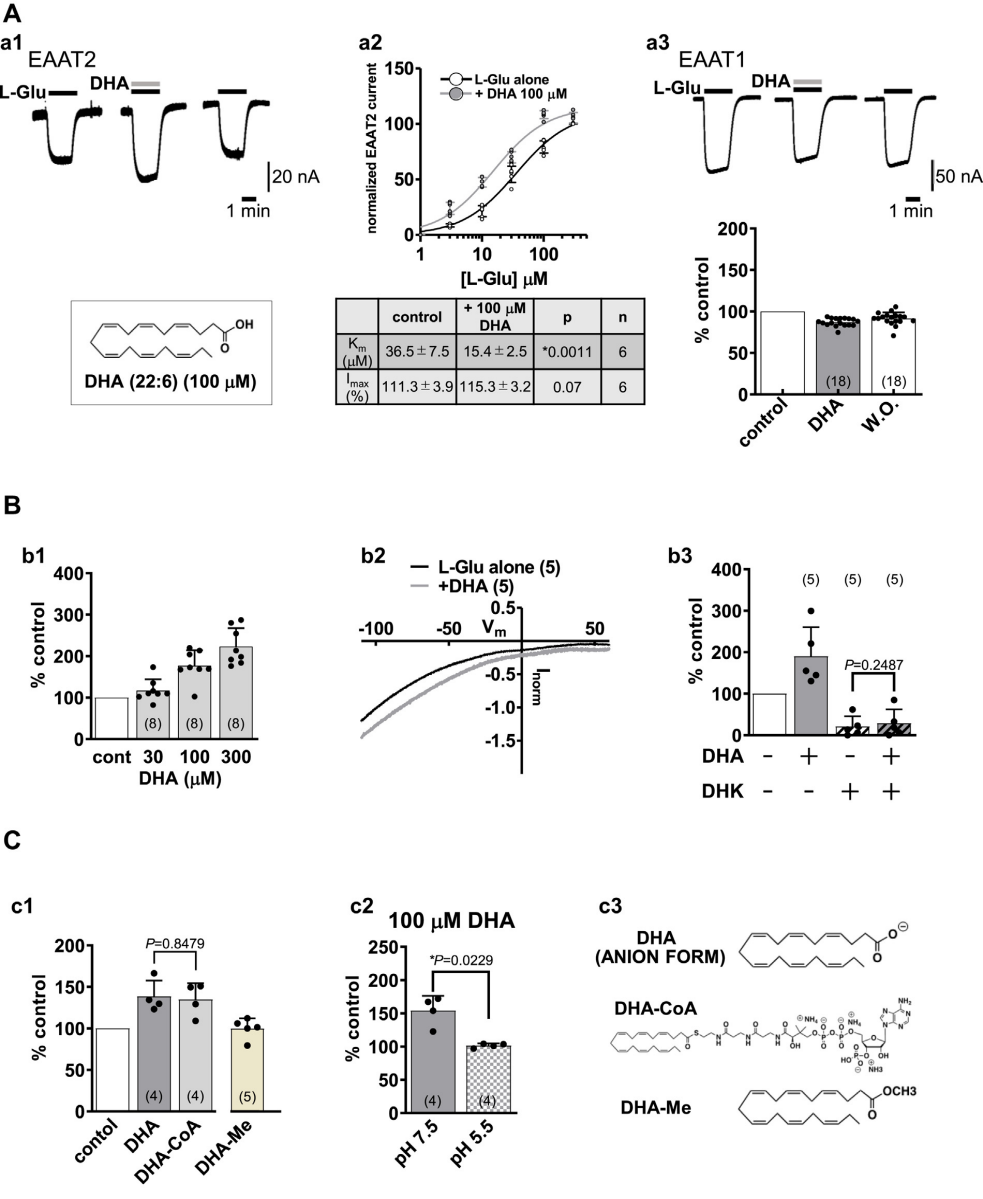
**Characterization of the effect of docosahexaenoic acid (DHA) on EAAT2 current.***A*, a1, representative traces of L-glutamate (L-Glu, 50 μM for 2 min, *black bar*)-induced current obtained from *Xenopus* oocytes over-expressing EAAT2 clamped at −50 mV in the absence or presence of DHA (100 μM for 2 min, *gray bar*). When co-applied, DHA increased the L-Glu-induced EAAT2 current, and the effect disappeared after washout. The structure of DHA is also shown. a2, effect of DHA (100 μM) on the L-Glu concentration–response curves of EAAT2 current at −50 mV. DHA caused a significant decrease of K_m_ for L-Glu, without significantly affecting the mean maximal current, I_max._ K_m_ is the Michaelis constant, which is L-Glu concentration needed to achieve a half-maximum binding at equilibrium. a3, representative traces of L-Glu (50 μM for 2 min, *black bars*)-induced EAAT1 current in the absence or presence of DHA (100 μM for 2 min, *gray bars*). When the compounds were co-applied, DHA tended to decrease EAAT1 current, and the effect disappeared after washout. Effects of DHA (100 μM) on the L-Glu-induced current amplitudes of EAAT1. The amplitudes were normalized to those just before the application of DHA. *B*, b1, concentration-dependence of the effect of DHA on L-Glu induced EAAT2 current. b2, current–voltage relationship for L-Glu-induced EAAT2 current in the absence or presence of DHA (100 μM). The effect of DHA was independent of holding potential. b3, in the presence of dihydrokainic acid (DHK, 100 μM), a selective inhibitor of EAAT2, DHA no longer enhanced the L-Glu-induced EAAT2 current. *C*, c1, comparison of the effects of DHA and that of DHA analog. The conjugate of DHA with coenzyme A (DHA-CoA, 100 μM), membrane-impermeable analog of DHA, increased the EAAT2 current to almost the same extent as DHA. DHA methyl ester (DHA-ME, 200 μM), an uncharged analog of DHA, had no effect on L-Glu-induced EAAT2 current. c2, the effect of DHA disappeared when the pH was changed from 7.5 to 5.5. c3, the structures of DHA and the analogs used in these experiments. Error bars represent mean ± SD. The numbers written within parentheses in each Figure represent the number of independent experiments. Statistical differences between groups were determined by two-tailed paired Student’s *t* test (a2, b3, c1, c2) (denoted by ∗). *p*-values are indicated in each Figure panel. EAAT1, excitatory amino acid transporter subtype 1.

We examined the effect of DHA on the L-Glu–induced current in *Xenopus* oocytes expressing EAAT2 by means of the two-electrode voltage-clamp method. We preliminarily confirmed that DHA alone did not induce any current (data not shown). As shown in the trace of EAAT2 current (Fig. 1*A*-a1), DHA (100 μM) increased the L-Glu (50 μM, 2 min)–induced EAAT2 current. When we examined the L-Glu concentration–response curve, DHA (100 μM) shifted the curve leftward (Fig. 1*A*-a2) with a significant decrease in K_m_ for L-Glu (K_m,_ control: 36.5 ± 7.5 μM; +DHA: 15.4 ± 2.5 μM; N = 6) without affecting in the mean maximal current (I_max_, control: 111.3 ± 3.9 %; +DHA: 115.3 ± 3.2 %; N = 6). K_m_ is the L-Glu concentration needed to achieve a half-maximum response at equilibrium. We also examined the effect of DHA on EAAT1, because its amino acid sequence is 65 % identical to EAAT2, and the degree of relatedness increases to 80 % when conservative substitutions are taken into account (6). In contrast to EAAT2, DHA tended to decrease EAAT1 current (Fig. 1*A*-a3). The effect of DHA (30–300 μM) on L-Glu–induced EAAT2 current was concentration dependent at −50 mV (Fig. 1*B*-b1) but was independent of holding potential (−110 mV to +60 mV) (N = 5) (Fig. 1*B*-b2). Subsequent experiments were therefore performed at the holding potential of −50 mV. DHA has been reported to modulate the activities of several ion channels (19, 20) that are endogenously expressed in *Xenopus* oocytes (21, 22, 23, 24). We therefore examined whether the effect of DHA was a direct effect on EAAT2 or not. As shown in Figure 1*B*-b3, dihydrokainic acid (DHK), a selective inhibitor of EAAT2 (6, 25), greatly reduced the L-Glu–induced current (21.0 ± 24.5 % of the control at 100 μM, N = 5), showing that the L-Glu-induced current in this experimental system is predominantly due to EAAT2. In the presence of DHK, DHA no longer enhanced the EAAT2 current (28.6 ± 33.9 % control, N = 5), indicating that the target of DHA is EAAT2. DHA has two main structural elements, a lipophilic acyl chain and a carboxyl group. A long lipophilic acyl chain might allow DHA to be incorporated into lipid bilayers. We therefore compared the effect of DHA with that of the conjugate of DHA with coenzyme A (DHA-CoA). Because CoA is a large hydrophilic moiety, DHA-CoA is membrane-impermeable and is unlikely to be incorporated into membranes (Fig. 1*C*-c3). As shown in Figure 1*C*-c1, 100 μM DHA-CoA increased the EAAT2 current to almost the same extent as DHA at the same concentration. We confirmed that CoA itself had no effect (104.0 ± 14.1 % control, N = 9). These results suggest that DHA does not interact with EAAT2 in membrane lipid bilayers or from the intracellular side but approaches from the outside of cells. A carboxylate is positioned at one end of DHA’s acyl chain and is deprotonated (negatively charged) at pH 7.5. DHA is reported to tune channel opening *via* electrostatic interaction between the negatively charged carboxylate and the channel’s voltage sensor (26, 27, 28, 29). To examine whether the negative charge of carboxylate is required for the DHA action, we examined the effect of DHA methyl ester (DHA-Me), an uncharged analog of DHA (Fig. 1*C*-c3). DHA-Me (200 μM) had no effect on EAAT2 current (Fig. 1*C*-c1), indicating that the carboxylate moiety is essential for the effects. When we changed the extracellular pH from 7.5 to 5.5, DHA had no effect on EAAT2 current. Because DHA is protonated and uncharged at pH 5.5, these data indicate the importance of the negative charge of carboxylate (Fig. 1*C*-c2). Considering that DHA-CoA, which has a negative charge derived from the phosphate group, also increased the EAAT2 current (Fig. 1*C*-c1, c3), these results suggest that the negative charge is important for the effect of DHA on EAAT2. ARA is reported to activate H^+^ conductance in EAAT4 (10, 30). The current–voltage relationships recorded in the presence of ARA intersected the control EAAT4 relationships and the crossing potential shifted markedly to the right as the extracellular pH was decreased (10, 30), meaning that ARA induces additional H^+^ current. However, DHA seems not to activate H^+^ conductance in the case of EAAT2 because the current–voltage relationships recorded in the presence of DHA at pH 7.5 did not intersect the control EAAT2 relationships from −110 mV to +60 mV (Fig. 1*B*-b2), and DHA had no effect on the EAAT2 current even at pH 5.5 at −50 mV (Fig. 1*C*-c2).

We also examined whether or not DHA metabolites contribute to the effect of DHA. In the ARA cascade, DHA is converted to maresin1 or neuroprotectin D1 by lipoxygenase (31). Neither nordihydroguaiaretic acid (NDGA, 100 μM), a lipoxygenase inhibitor, nor indomethacin (Indo, 100 μM), a cyclooxygenase inhibitor, affected the DHA-induced enhancement of EAAT2 current (Fig. S1*A*). To examine the contribution of the hydrophilicity (polarity) of DHA (32), we examined the action of DHA-ethanolamide (DHA-EA), an uncharged hydrophilic analog of DHA, and DHA-EA (100 μM) had little effect on the EAAT2 current (Fig. S1*B*).

### Identification of the target site of DHA on EAAT2

**Figure 2:**
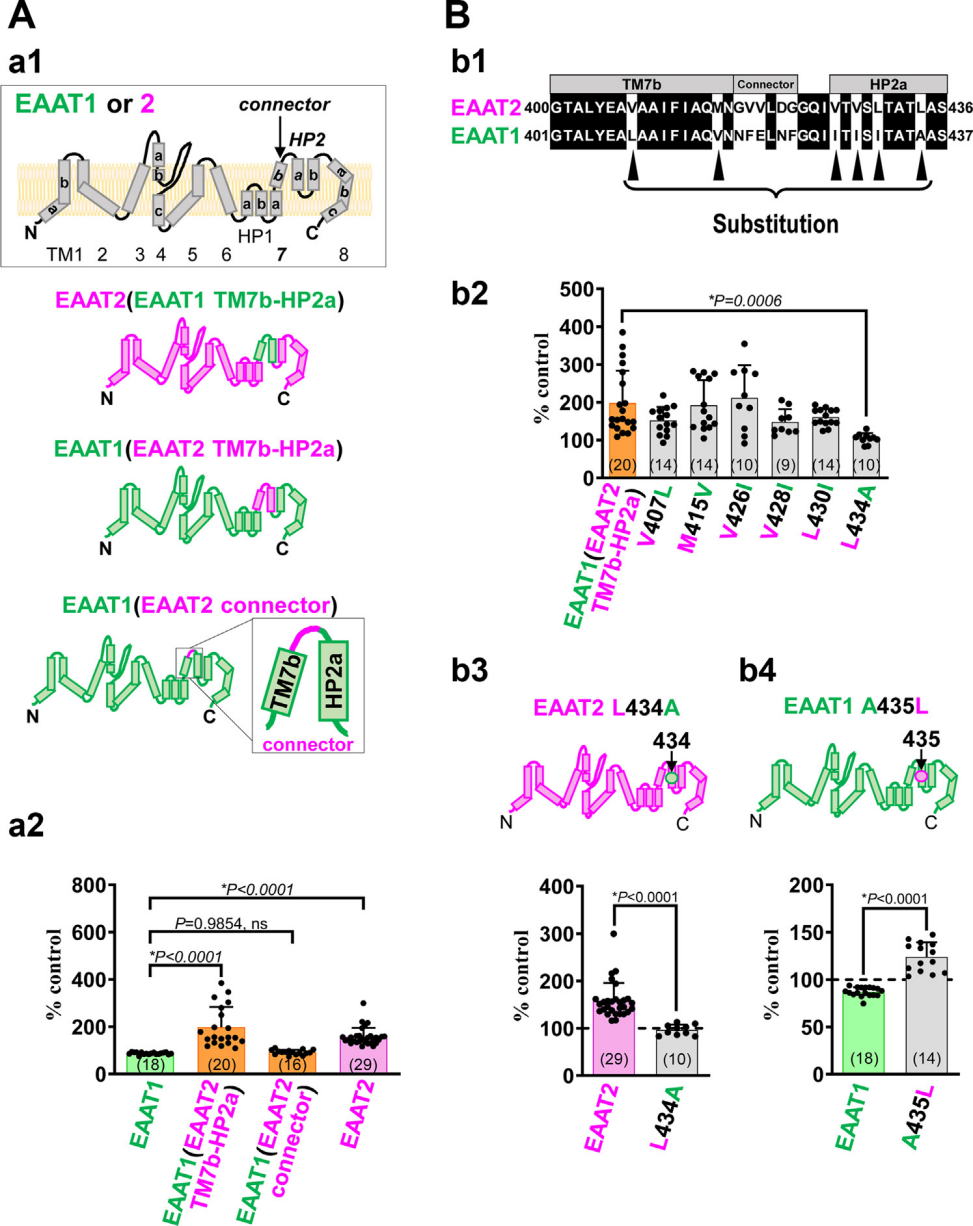
**Leu434 residue in re-entrant hairpin loops HP2a is essential for the augmenting effect of DHA on L-Glu-induced EAAT2 current.***A*, a1, topology of EAAT1, EAAT2, and EAAT1-EAAT2 hybrid chimeras: EAAT2(EAAT1 TM7b-HP2a), EAAT1(EAAT2 TM7b-HP2a), and EAAT1(EAAT2 connector). a2, the effect of DHA on L-Glu-induced currents of EAAT1, EAAT1(EAAT2 TM7b-HP2a), EAAT1(EAAT2 connector), and EAAT2. Data are shown as rates of increase by DHA. *B*, b1, amino acid alignment from TM7b to HP2a of EAAT2 and EAAT1. The common amino acids are shown on a *black background*. Single amino acid back mutations were performed at the sites indicated by *black arrowheads* in EAAT1(EAAT2 TM7b-HP2a) chimera. b2, comparison of the effects of DHA on EAAT1(EAAT2 TM7b-HP2a) chimera and a series of EAAT1(EAAT2 TM7b-HP2a)s with point back mutations to the original amino acid of EAAT1 for the six amino acids shown in b1. Only EAAT1(EAAT2 TM7b-HP2a) L434A shows complete loss of the augmenting effect of DHA. Data are shown as rates of increase by DHA. Exact *p*-values were 0.7809 for V407L, 0.1898 for M415V, 0.1575 for V426I, 0.4775 for V428I, 0.3042 for L430I, and 0.0006 for L434A. *versus* EAAT1(EAAT2 TM7b-HP2a group). b3, (*top*) topology of EAAT2 L434A; (*bottom*) comparison of the effects of DHA on EAAT2 and EAAT2 L434A. Data are shown as rates of increase by DHA. b4, (*top*) topology of EAAT1 A435L; (*bottom*) comparison of the effects of DHA on EAAT1 and EAAT1 A435L. Data are shown as rates of increase by DHA. Error bars represent mean ± SD. The numbers written within parentheses in each Figure represent the number of independent experiments. Statistical differences between groups were determined by two-tailed unpaired Student’s *t* test (b3 and b4), and Tukey’s test following one-way factorial ANOVA (a2 and b2) (denoted by ∗). *p*-values are indicated in each Figure panel. DHA, docosahexaenoic acid; EAAT1, excitatory amino acid transporter subtype 1; EAAT2, excitatory amino acid transporter subtype 2.

As shown in Figure 1*A*-a2 and a3, DHA significantly increased EAAT2 current, while it slightly decreased EAAT1 current. We next focused on this difference. Transmembrane regions 7b—re-entrant hairpin loops 2a (TM7b-HP2a) in the transport domain (Fig. 2*A*-a1) was shown to be responsible for the difference in the EC_50_ values of EAAT1 and EAAT2 (33). We therefore speculated that TM7b-HP2a (Val407-Leu434 in EAAT2 numbering) is involved in the difference between the effects of DHA on EAAT1 and EAAT2. We first employed EAAT2(EAAT1 TM7b-HP2a), a chimera in which the TM7b-HP2a region of EAAT2 is substituted by the corresponding region of EAAT1. However, this chimera was nonfunctional. We therefore used an EAAT1-based chimera, EAAT1(EAAT2 TM7b-HP2a), in which the EAAT1 TM7b-HP2a region is substituted by that of EAAT2. In this chimera, the current was enhanced by DHA (198.5 ± 85.1 % control, N = 20) (Figs. 2*A*-a2 and S3). The extracellular domain between TM7b and HP2a (the ‘connector’: Gly417-Gly422 in EAAT2 numbering) is the least-conserved motif between EAAT1 and EAAT2 in TM7b-HP2a. However, replacement of the EAAT1 ‘connector’ by the EAAT2 ‘connector’ did not alter the effect of DHA (92.3 ± 10.4 % control, N = 16) (Figs. 2*A*-a2 and S3), indicating that the ‘connector’ motif is not involved in the effect of DHA. From TM7b to HP2a but connector region, there are six different amino acids between EAAT2 and EAAT1 (Val407, Met415, Val426, Val428, Leu430, and Leu434 in EAAT2) (black arrowheads in Figs. 2*B*-b1 and S2). To identify the essential amino acid(s), we constructed EAAT1(EAAT2 TM7b-HP2a) containing point mutations (back-mutation to the original EAAT1 amino acid residue) in the six amino acids described above (V407L, M415V, V426I, V428I, L430I, and L434A) (Fig. 2*B*-b2). Among these mutants, only EAAT1(EAAT2 TM7b-HP2a) L434A showed complete disappearance of the effect of DHA (Figs. 2*B*-b2 and S3), indicating that EAAT2 Leu434 is the essential. To confirm this, we mutated Leu434 in EAAT2 to Ala (EAAT2 L434A), because Ala435 is the EAAT1 residue corresponding to Leu434 of EAAT2. Indeed, in EAAT2 L434A, the effect of DHA was completely abolished (from 158.1 ± 37.9% to 96.5 ± 11.2%) (Figs. 2*B*-b3 and S3). On the other hand, when we mutated EAAT1 Ala435 to Leu, DHA enhanced the EAAT1 A435L current (from 87.1 ± 4.8 % to 124.0 ± 15.4 %) (Figs. 2*B*-b4 and S3). These results confirm that EAAT2 Leu434 is responsible for the augmenting effect of DHA on EAAT2 current, and the absence of Leu at the corresponding position of EAAT1 accounts for its lack of response to DHA.

The net L-Glu–induced EAAT currents is composed of the coupled L-Glu transport currents and the uncoupled Cl^−^ (anion) currents (16). EAAT2 is a very effective transporter with predominant L-Glu transport currents, so that anion currents may not have major contributions to the EAAT2 currents (16), while in case of EAAT1, anion currents voltage-dependently contribute to the total EAAT1 currents. We therefore confirmed whether or not DHA regulate L-Glu transport currents and anion currents distinctly focusing in EAAT1, EAAT1-based single mutants and chimeras. When extracellular Cl^−^ is replaced by NO^3−^, which is about 17 times more permeable than Cl^−^ (34), the anion current becomes pronounced in EAAT1 currents. In NO^3−^-based buffer, current-voltage relationships for EAAT1 current in the absence or presence of DHA (100 μM) were almost same (Fig. S4*A*-a1). The measurement at +60 mV in NO^3−^-based buffer allows us to isolate changes in the anion current. At +60mV, DHA had no effects on the anion current (Fig. S4*A*-a2). Similar results were obtained in both cases of EAAT1(EAAT2 TM7b-HP2a) and EAAT1 A435A (Fig. S4, *B* and *C*). These results indicates that DHA does not regulate anion currents of EAAT1.

### Identification of PUFAs that also augment EAAT2 current

**Figure 3:**
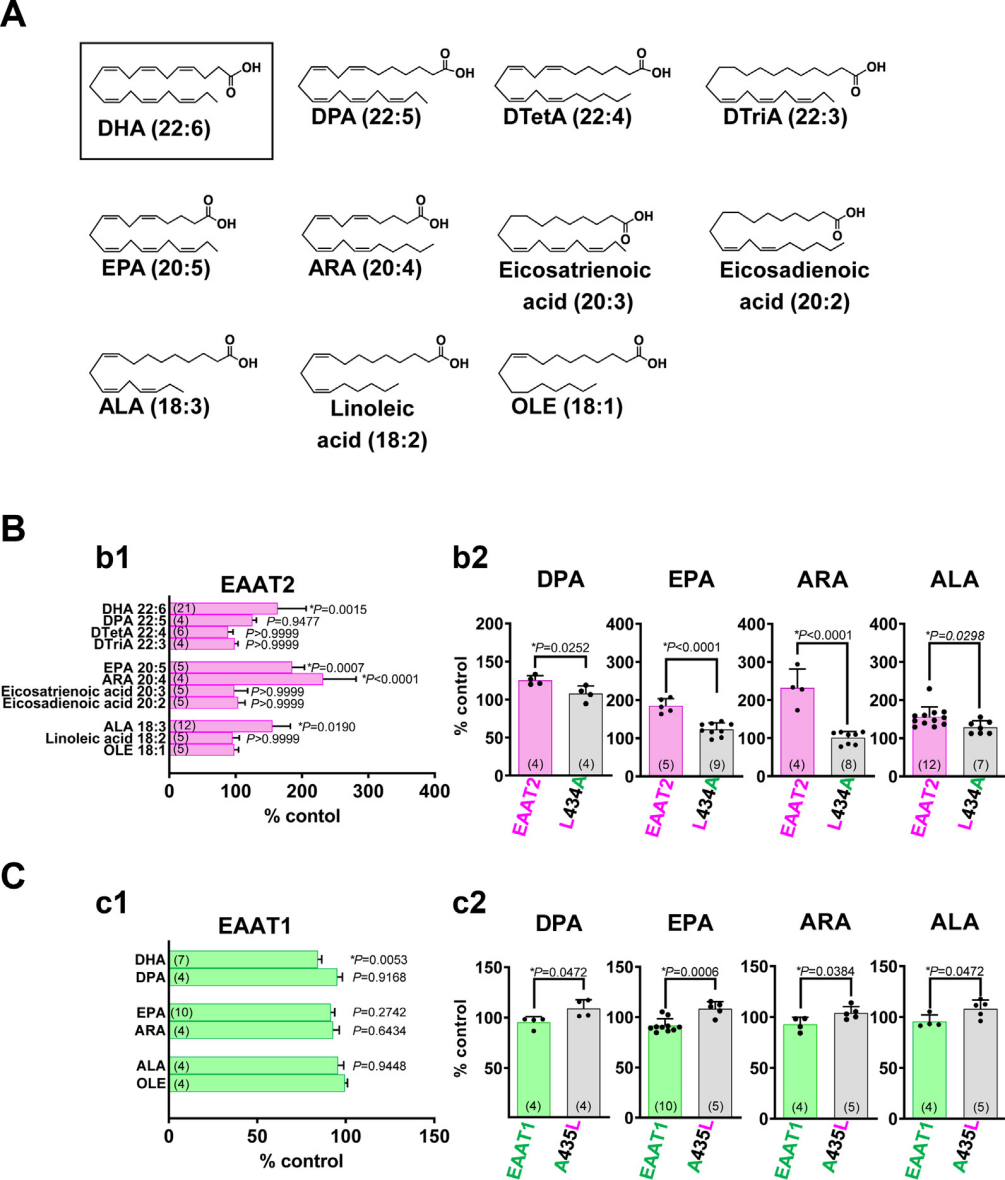
**Identification of other PUFAs that augment L-Glu-induced EAAT2 current.***A*, structures of fatty acids used in this experiment. ALA, α-linolenic acid; ARA, arachidonic acid; DHA, docosahexaenoic acid; DPA, docosapentaenoic acid, DTetA, docosatetraenoic acid, DTriA, docosatrienoic acid, EPA, eicosapentaenoic acid; OLE, oleic acid. *B*, b1, effects of fatty acids (100 μM) shown in *A* on the L-Glu-induced EAAT2 current. DHA, DPA, EPA, ARA, and ALA significantly increased the current. b2, loss of the augmenting effects of DPA, EPA, ARA and ALA in EAAT2 L434A. *C*, c1, effects of DHA, DPA, EPA, ARA, and ALA (100 μM) on the L-Glu-induced EAAT1 current. DHA significantly decreased the current, while DPA, EPA, ARA, and ALA tended to decrease the current. c2, the effects of DPA, EPA, ARA, and ALA on L-Glu-induced EAAT1 A435L current. These PUFAs augmented the L-Glu-induced EAAT1 A435L current. Error bars represent mean ± SD. The numbers written within parentheses in each Figure represent the number of independent experiments. Statistical differences between groups were determined by two-tailed unpaired Student’s *t* test (b2 and c2), and Tukey’s test following one-way factorial ANOVA (b1 and c1 *versus* OLE-treated group) (denoted by ∗). *p*-values are indicated in each Figure panel. EAAT2, excitatory amino acid transporter subtype 2; PUFA, polyunsaturated fatty acid.

Some functional proteins are modulated by multiple PUFAs including DHA (35). We therefore examined the effects of 10 fatty acids with various acyl chain lengths and various numbers of unsaturated bonds (Fig. 3*A*). Among these fatty acids, docosapentaenoic acid (DPA), eicosapentaenoic acid (EPA), ARA, and α-linolenic acid (ALA) augmented EAAT2 current similarly to DHA (125.3 ± 6.0%, 185.2 ± 18.6%, 232.0 ± 49.4%, and 155.6 ± 26.9%, respectively, *versus* the oleic acid-treated group) (Fig. 3*B*-b1). To examine the involvement of EAAT2 Leu434 in these effects, we applied DPA, EPA, ARA, and ALA to EAAT2 L434A (Fig. 3*B*-b2). Notably, the effects of these PUFAs were completely abrogated by the single mutation. We also examined the effects of DPA, EPA, ARA, and ALA on EAAT1 current (Fig. 3*C*-c1). DPA, EPA, ARA, and ALA tended to decrease the EAAT1 current. The effects of these PUFAs on EAAT1 were also changed to an augmenting effect by single mutation of EAAT1 Ala435 to Leu (Fig. 3*C*-c2). These data demonstrate the importance of EAAT2 L434 and the corresponding amino acid of EAAT1 in the effects of PUFAs on L-Glu transport.

### Docking simulation of DHA and EAAT2

**Figure 4:**
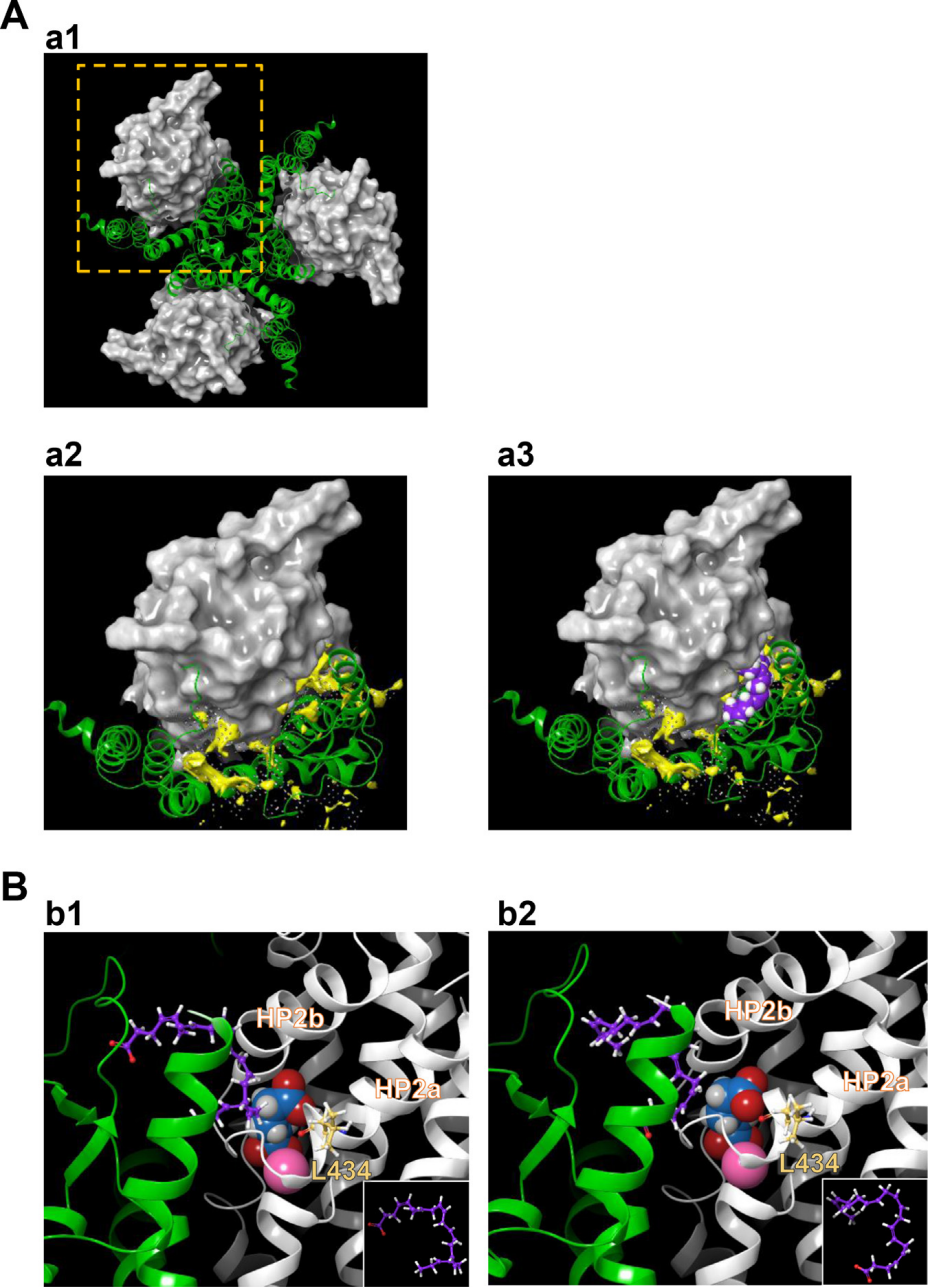
**Proposed binding conformation for DHA in the transport/trimerization domain interface of EAAT2 homology model in the outward facing state OFS.***A*, a1, extracellular view of trimerized EAAT2 OFS homology model based on EAAT1 crystal structure. Trimerization domain is shown in *green ribbon*. Transporter domain is shown in *gray surface*. Structural data were presented using graphical user interface in Maestro Suite. The homology model of EAAT2 was constructed as a monomer based on the crystal structure of OFS EAAT1 (PDBID: 5LLM) with energy-based loop refinement using Homology Modeling unit in Maestro Suite. The quality of homology model was checked by PROCHECK. a2 and a3, magnified monomer in the hatched square in a1 in the absence (a2) or presence (a3) of DHA. The lipid crevice calculated by SiteMap exists at the interface between trimerization domains and transport domains (*yellow space*) (a2). DHA is docked to the lipid crevice (carbon: *purple spheres*; hydrogen: *white spheres*) (a3). *B*, b1 and b2, docking poses of DHA to the lipid pocket in the vicinity of HP2 according to induced fit docking protocol. The trimerization domain and transport domain are shown in *green and gray ribbons*, respectively. Carbons in DHA and EAAT2 L434 are represented by *purple and yellow sticks*, respectively. The atoms in L-Glu are shown as follows: carbon: *blue sphere*; hydrogen: *white sphere*, oxygen: *red sphere*; nitrogen: hiding. Na^+^ is shown as a *pink sphere*. Two types of the DHA conformations could be visualized according to the position of the carboxylic group, *i.e.*, one is with carboxyl group on upper side (b1) and the other is with carboxyl group on lower side (b2). Both of them have similar U-shaped conformation. Inset is the DHA conformations in each case. Three-dimensional position of DHA is in close proximity to L-Glu binding site and Na^+^ binding site. DHA, docosahexaenoic acid; EAAT2, excitatory amino acid transporter subtype 2; OFS, outward facing state.

To visualize the positional relationships between DHA and EAAT2 Leu434, we performed induced fit docking (IFD) simulation (36, 37, 38) (Figs. 4 and S5*B*-b1 and b2). Although the crystal structure of EAAT2 has not been reported yet, that of EAAT1 was clarified in 2017 (protein data bank ID [PDBID]: 5LLM) (39), so we performed the standard IFD simulation of DHA against EAAT2 homology model using EAAT1 as a template (Fig. S5*A*). In addition to the crevice for allosteric inhibitor UCPH_101_, the second hydrophobic crevice was found in EAAT1 between TM4 and HP2 (39), which is on the extracellular half of transport/trimerization domain interface. According to our data so far, we assumed this site to be the centroid for interaction with DHA. The structural changes during substrate transport have been studied in detail by modeling Glt_Ph_ (the EAAT homolog of archaea [*Pyrococcus horikoshii*]): outward facing state (OFS) (40, 41),→intermediate (i) OFS (42)→unlocked inward facing state (IFS) (43)→IFS (44). In OFS of EAAT2, DHA could occupy the hydrophobic crevice (Fig. 4*A*-a1–a3). A standard IFD protocol (Glide, Schrödinger, LLC, New York, NY, 2019; Prime, Schrödinger, LLC, New York, NY, 2019) afforded seven candidate combinations of docking site and DHA pose. The two top-scoring combinations are shown in Figure 4*B*-b1 and b2. For methodological details, see “Structural data, presentation, and molecular modeling studies” in Experimental procedure. In both cases, DHA was located in close proximity to the L-Glu binding site and Na^+^ binding site (Fig. 4*B*), and the DHA poses were U-shaped (Fig. 4*B*-b1 and b2, inset).

## Discussion

It has been proposed that the lipophilic PUFA tails show nonspecific hydrophobic interactions, while the carboxylic head group displayed more specific interactions (45, 46, 47). The pH dependence of DHA’s activity suggests that the negative charge of its carboxylate moiety is important for the current action. DHA is reported to modulate voltage-gated Shaker K^+^ channel activity (26, 27, 28) *via* a lipoelectric mechanism. In case of Shaker, carboxylic head group interacts with six positively charged amino acid residues in the voltage sensor domain (29). The lipoelectric mechanism may be involved in the interaction between DHA and EAAT2 as well. Docking simulation indicated the carboxylate moiety of DHA was close to the positively charged amino acid residues of EAAT2 in OFS. Because the elevator-like motion of EAAT2 transport is dominated by the positive charge of the bound Na^+^ (48, 49, 50, 51), it is possible that the negative charge of DHA could stabilize OFS, resulting in efficient binding of Na^+^ and substrate. We also found DHA (22:6), DPA (22:5), EPA (20:5), ARA (20:4), and ALA (18:3) had the same activity as DHA. The effects are not simply correlated with the number of unsaturated bonds in the acyl chain, because among ALA (18:3), eicosatrienoic acid (20:3), and DTriA (22:3), only ALA augmented EAAT2 current. The occupancy of unsaturation bonds in the acyl chain and the resulting conformations may be related to the effects. Our docking simulation between DHA and homology EAAT2 OFS suggests the U-shaped conformation is important to stay in the hydrophobic crevice.

The crystal structure of Glt_Ph_ (41, 42, 43), the EAAT homolog of archaea [*P. horikoshii*] and its structural transition during the transport process have been investigated in detail. Each protomer of homotrimer is comprised of eight TM helices and two re-entrant HPs (40). The protomer is divided into two distinct functional components: one is a rigid scaffold domain that mediates interprotomer interactions and is located in the center of the trimer and the other is a transport domain containing the substrate-binding site (43). There are hydrophobic crevices with strong nonprotein electron density in the OFS and unlocked IFS (41, 43). In the presences of substrate and Na^+^, the Glt_Ph_ lipid pocket in this hydrophobic crevice includes 12 amino acid residues (Val86, Leu90, Pro128, Leu130, Ile133, Leu134, Ile137, Leu152, Ile309, Leu347, Ile350, and Gly351, see Fig. S2, pink background) (PDBID: 2NWL) (41). Among these amino acids, Glt_Ph_ Leu347 is corresponding to EAAT2 Leu434. Substrate binding induces sliding of the transport domain from the outside to the inside of the membrane, in an ‘elevator-like’ motion (44, 52) and changes the interface space between the transport domain and the scaffold domain (53). Mutant Glt_Ph_ bearing two “humanizing” mutations, R276S/M395R, enhanced ‘elevator-like’ dynamics and substrate transport rate (43). These two amino acids locate in the lipid crevice, indicating the importance of unlocking of the transport domain and the elevator-like slide for transport cycle. Docking simulation of Glt_Ph_ and palmitic acid (16:0) shows that palmitic acid enters the lipid pocket in the crevice (PDBID: 2NWX) (41). Considering that the augmenting effect of DHA on EAAT2 current was abrogated by L434A substitution, while the effect of DHA was switched from inhibition to augmentation in EAAT1 A435L, the difference between Leu and Ala seems to be important for the augmentative effect of DHA. The side chains of Leu and Ala are isobutyl and methyl, respectively, and the difference in the amino acid surface areas might be critical for the effect. Leu434 is located in HP2, at the surface of the lipid crevice. HP2 is important for the elevator-like slide (43), and our docking simulation indicated that DHA is docked to the lipid crevice at the transport/trimerization domain interface, in close proximity to the L-Glu binding site and the Na^+^ binding site. These data suggest that the interaction between DHA and the hydrophobic pocket involving Leu434 could affect the elevator-like slide and also the shapes of the L-Glu binding site and the Na^+^ binding site, thereby enhancing transport efficiency. It has been reported that K^+^ also binds to Na^+^ binding site (54) during retranslocation. Since K^+^-bound retranslocation is reported to be rate-limiting for mammalian EAATs (18, 55), it is possible that DHA may influence the K^+^-bound retranslocation process.

This study show that DHA augments the L-Glu-induced EAAT2 current, and the Leu434 residue in the HP2a is essential for this activity. Furthermore, our data suggest that EAAT2 Leu434 and EAAT1 Ala435 (the amino acid residue corresponding to EAAT2 Leu434) are important for the effects of PUFAs on EAATs. Our findings would become a foothold to investigate the mechanisms underlying the interactions between PUFAs and EAATs.

## Experimental procedures

### Molecular biology

pcDNA3.1 containing cDNA of human brain EAAT1 (GeneBank accession no. D26443) or EAAT2 (GeneBank accession no. D85884.1) was kindly provided by Dr Keiko Shimamoto (Bioorganic Research Institute, Suntory Foundation for Life Sciences). The chimeric cDNAs used in the preliminary experiments were kind gifts from Dr Robert J. Vandenberg (The University of Sydney, Australia). EAAT1(EAAT2 TM7b-HP2a), EAAT2(EAAT1 TM7b-HP2a), and EAAT1(EAAT2 connector) were constructed by means of splicing overlap extension PCR (56). Point mutations were induced in our lab using a KOD-Plus-Mutagenesis kit (Toyobo) according to the manufacturer’s instructions. The sequence was confirmed for each construction (Macrogen-Japan). The cDNAs were digested with *BamH1* and *Xba1* and subcloned into pGEMHE vector, which was a kind gift from Dr Yoshihiro Kubo (National Institute for Physiological Sciences). The coding sequence was inserted between *Xenopus β*-globulin 5′- and 3′-untranslated regions (57) in the plasmid, which had been linearized by cutting at the *Sph1* site. The capped cRNA was synthesized from the linearized cDNA using a mMESSAGE mMACHINE kit (Ambion) according to the manufacturer’s instructions.

### Expression of EAATs in Xenopus oocytes

Animal experiments were carried out in accordance with the principles of the Basel Declaration and the recommendations of the Guide for the Care and Use of Laboratory Animals, the Animal Research Committee of the National Institute of Health Sciences, Japan. The protocol was approved by the Animal Research Committee of the National Institute of Health Sciences, Japan. Oocytes were collected from anesthetized *Xenopus laevis*. The isolated oocytes were then treated with type1 collagenase (2 mg ml^−1^, Sigma) and injected with 50 nl of capped cRNA solution (>10 ng), after which they were incubated for 2 to 4 days at 18 °C in ND96 (NaCl 96, KCl 2, CaCl_2_ 1.8, MgCl_2_ 1, Hepes 5 [mM], pH 7.5) supplemented with 0.01 % gentamycin.

### Electrophysiology

Two-electrode voltage clamp recordings from the oocytes were performed at room temperature (25 °C) using glass microelectrodes filled with 3 M KCl (resistance = 1–4 MΩ) and an Ag/AgCl pellet electrode (EP2; World Precision Instruments). A bath-clamp amplifier (OC-725C; Warner Instruments) was used with a Digidata 1320A interface (Axon Instruments). The pClamp software suite (ver. 8.2; Axon Instruments) and the Clampfit data acquisition software were used for stimulation control, data acquisition, and data analysis. The oocytes were clamped at −50 mV and L-Glu–induced current was measured under continuous superfusion with ND96. In the pharmacological experiments, fatty acids or antagonists were co-applied with L-Glu. The normalized mean concentration response curves of currents induced by L-Glu at 300 μM were fitted by nonlinear regression analysis to the equation I = I_max_([L-Glu]/[L-Glu] + K_m_), where I_max_ is the maximal current and K_m_ is the Michaelis constant, which is the L-Glu concentration needed to achieve a half-maximum response at equilibrium. The K_m_ value was determined by fitting results from individual saturation response curves. To examine the current–voltage relationship, L-Glu–induced current was calculated by subtraction of the steady-state current from the L-Glu-induced current. The curves were obtained with a holding potential of −60 mV applying 8000 ms ramp pulse from −110 to +60 mV. Data are shown as the values normalized to that obtained with 50 μM L-Glu at –100 mV. In the experiments performed at pH 5.5, Hepes was replaced by 2-(N-morpholino)ethanesulfonic acid to adjust the extracellular pH. In the experiments to substitute nitrate (NO^3−^) for Cl^−^, 96 mM NaNO_3_ was used instead of NaCI, and Cl^−^ was replaced by an equimolar gluconate ion. NO^3−^-based ND96 was contained in NaNO_3_ 96, K-gluconate 2, Ca-gluconate 1.8, Mg-gluconate 1, and Hepes 5 [mM] at pH 7.5.

### Structural data, presentation, and molecular modeling studies

Structural data were presented using the graphical user interface in Maestro Suite (Schrödinger, LLC,). The homology model of EAAT2 was constructed as a monomer based on the crystal structure of OFS EAAT1 (PDBID: 5LLM) with energy-based loop refinement using the Homology Modeling unit in Maestro Suite (Schrödinger, LLC). The quality of the homology model was checked by PROCHECK as shown by a Ramachandran plot (58, 59) (Fig. S5). The red regions are the “most favored” regions, the yellow regions are the “additionally allowed” regions, the thin yellow regions are the “generously allowed” regions, and the white regions are the “disallowed” regions.

The homology model used for docking contains bound ligand, L-aspartate, which was replaced by the substrate in EAAT2 (L-Glu) before minimization was conducted. The co-crystalized Na^+^ was retained, and the whole complex was minimized locally using Prime (Prime, Schrödinger, LLC). Possible ligand binding sites were detected by SiteMap (SiteMap, Schrödinger, LLC), and the docking grid of DHA was determined as the hydrophobic site near the transport/trimerization domain interface. We docked DHA, preprepared using LigPrep to obtain its ionized form, to the docking grid of the EAAT2 homology model by using a standard IFD protocol (Glide, Schrödinger, LLC; Prime, Schrödinger, LLC). As a result, seven poses were generated, and among them, the two docking poses having the top IFD scores showed similar conformations but with different orientations of the carbonyl group.

### Statistical analysis

Statistical analysis was performed using GraphPad Prism 9 (Ver. 9, GraphPad software). All data are shown as mean ± SD. Statistical analyses were performed using Tukey’s test following one-way factorial ANOVA or two-tailed Student’s *t* test. Differences were considered significant at *p* < 0.05. Statistical test, the number of independent experiments, and *p* values are indicated in each Figure panel or legend.

### Materials

All chemical compounds were purchased from Wako unless otherwise stated. DHA (cis-4,7,10,13,16,19-docosahexaenoic acid: DHA), DHA-Me, ARA, EPA (cis-5,8,11,14,17-eicosapentaenoic acid), DHK, indomethacin, and Triton X-100 were purchased from Sigma. DHA-CoA and CoA were purchased from Avanti. Oleic acid, linoleic acid, ALA, cis-11,14-eicosadienoic acid, cis-11,14,17-eicosatrienoic acid, cis-13,16,19-docosatrienoic acid (DTriA), cis-7,10,13,16-docosatetraenoic acid (DTetA), cis-7,10,13,16,19-docosapentaenoic acid (DPA), and N-(2-hydroxyethyl)-4Z,7Z,10Z,13Z,16Z,19Z-docosahexaenamide (DHA-EA) were purchased from Cayman Chemical. L-Glu stock solution (20 mM) in purified water (DIRECT-Q; Millipore) was diluted to the required final concentration with ND96 just before application. Stock solutions of fatty acids (100 mM) in ethanol was diluted to the required final concentration with ND96 on demand and used within 2 h to avoid precipitation. We confirmed that the vehicle had no effect prior to each experiment.

## Data availability

All data other than those in the following public data base are contained within the manuscript.

GenBank No.

**Table:** 

Transporter	Accession numbers
EAAT1	D26443.1
EAAT2	D85884.1
Gltph	NP_143181

Protein Data Bank (PDB) No.

**Table:** 

Transporter	Accession numbers
EAAT1	5LLM
Gltph	2NWX

## Supporting information

This article contains supporting information (41, 58, 59).

## Conflicts of interest

The authors declare that they have no conflicts of interest with the contents of this article.

## References

[1] Fairbairn P., Tsofliou F., Johnson A., Dyall S.C. (2019). Combining a high DHA multi-nutrient supplement with aerobic exercise: protocol for a randomised controlled study assessing mobility and cognitive function in older women. Prostaglandins Leukot. Essent. Fatty Acids.

[2] McNamara R.K., Kalt W., Shidler M.D., McDonald J., Summer S.S., Stein A.L. (2018). Cognitive response to fish oil, blueberry, and combined supplementation in older adults with subjective cognitive impairment. Neurobiol. Aging.

[3] Yurko-Mauro K., Alexander D.D., Van Elswyk M.E. (2015). Docosahexaenoic acid and adult memory: a systematic review and meta-analysis. PLoS One.

[4] Wurtman R.J. (2017). Synapse formation in the brain can be enhanced by co-administering three specific nutrients. Eur. J. Pharmacol..

[5] Cao D., Kevala K., Kim J., Moon H.-S., Jun S.B., Lovinger D. (2009). Docosahexaenoic acid promotes hippocampal neuronal development and synaptic function. J. Neurochem..

[6] Arriza J.L., Fairman W.A., Wadiche J.I., Murdoch G.H., Kavanaugh M.P., Amara S.G. (1994). Functional comparisons of three glutamate transporter subtypes cloned from human motor cortex. J. Neurosci..

[7] Arriza J.L., Eliasof S., Kavanaugh M.P., Amara S.G. (1997). Excitatory amino acid transporter 5, a retinal glutamate transporter coupled to a chloride conductance. Proc. Natl. Acad. Sci. U. S. A..

[8] Fairman W.A., Vandenberg R.J., Arriza J.L., Kavanaugh M.P., Amara S.G. (1995). An excitatory amino-acid transporter with properties of a ligand-gated chloride channel. Nature.

[9] Zerangue N., Arriza J.L., Amara S.G., Kavanaugh M.P. (1995). Differential modulation of human glutamate transporter subtypes by arachidonic acid. J. Biol. Chem..

[10] Fairman W.A., Sonders M.S., Murdoch G.H., Amara S.G. (1998). Arachidonic acid elicits a substrate-gated proton current associated with the glutamate transporter EAAT4. Nat. Neurosci..

[11] Berry C.B., Hayes D., Murphy A., Wiessner M., Rauen T., McBean G.J. (2005). Differential modulation of the glutamate transporters GLT1, GLAST and EAAC1 by docosahexaenoic acid. Brain Res..

[12] Danbolt N.C. (2001). Glutamate uptake. Prog. Neurobiol..

[13] Zerangue N., Kavanaugh M.P. (1996). Flux coupling in a neuronal glutamate transporter. Nature.

[14] Holmseth S., Dehnes Y., Huang Y.H., Follin-Arbelet V.V., Grutle N.J., Mylonakou M.N. (2012). The density of EAAC1 (EAAT3) glutamate transporters expressed by neurons in the mammalian CNS. J. Neurosci..

[15] Tanaka K., Watase K., Manabe T., Yamada K., Watanabe M., Takahashi K. (1997). Epilepsy and exacerbation of brain injury in mice lacking the glutamate transporter GLT-1. Science.

[16] Wadiche J.I., Amara S.G., Kavanaugh M.P. (1995). Ion fluxes associated with excitatory amino acid transport. Neuron.

[17] Levy L.M., Warr O., Attwell D. (1998). Stoichiometry of the glial glutamate transporter GLT-1 expressed inducibly in a Chinese hamster ovary cell line selected for low endogenous Na+-dependent glutamate uptake. J. Neurosci..

[18] Vandenberg R.J., Ryan R.M. (2013). Mechanisms of glutamate transport. Physiol. Rev..

[19] Boland L.M., Drzewiecki M.M. (2008). Polyunsaturated fatty acid modulation of voltage-gated ion channels. Cell Biochem. Biophys..

[20] Mayol V., Duran M.J., Gerbi A., Dignat-George F., Lévy S., Sampol J. (1999). Cholesterol and omega-3 fatty acids inhibit Na, K-ATPase activity in human endothelial cells. Atherosclerosis.

[21] Barish M.E. (1983). A transient calcium-dependent chloride current in the immature Xenopus oocyte. J. Physiol..

[22] Dascal N., Snutch T.P., Lubbert H., Davidson N., Lester H.A. (1986). Expression and modulation of voltage-gated calcium channels after RNA injection in Xenopus oocytes. Science.

[23] Miledi R. (1982). A calcium-dependent transient outward current in Xenopus laevis oocytes. Proc. R. Soc. Lond. B Biol. Sci..

[24] Englund U.H., Gertow J., Kågedal K., Elinder F. (2014). A voltage dependent non-inactivating Na+ channel activated during apoptosis in Xenopus oocytes. PLoS One.

[25] Robinson M.B., Hunter-Ensor M., Sinor J. (1991). Pharmacologically distinct sodium-dependent L-[3H]glutamate transport processes in rat brain. Brain Res..

[26] Borjesson S.I., Elinder F. (2011). An electrostatic potassium channel opener targeting the final voltage sensor transition. J. Gen. Physiol..

[27] Borjesson S.I., Hammarstrom S., Elinder F. (2008). Lipoelectric modification of ion channel voltage gating by polyunsaturated fatty acids. Biophysical J..

[28] Borjesson S.I., Parkkari T., Hammarstrom S., Elinder F. (2010). Electrostatic tuning of cellular excitability. Biophys. J..

[29] Borjesson S.I., Elinder F. (2008). Structure, function, and modification of the voltage sensor in voltage-gated ion channels. Cell Biochem. Biophys..

[30] Tzingounis A.V., Lin C.L., Rothstein J.D., Kavanaugh M.P. (1998). Arachidonic acid activates a proton current in the rat glutamate transporter EAAT4. J. Biol. Chem..

[31] Bazinet R.P., Laye S. (2014). Polyunsaturated fatty acids and their metabolites in brain function and disease. Nat. Rev. Neurosci..

[32] Kawanabe A., Okamura Y. (2016). Effects of unsaturated fatty acids on the kinetics of voltage-gated proton channels heterologously expressed in cultured cells. J. Physiol..

[33] Mitrovic A.D., Amara S.G., Johnston G.A., Vandenberg R.J. (1998). Identification of functional domains of the human glutamate transporters EAAT1 and EAAT2. J. Biol. Chem..

[34] Wadiche J.I., Kavanaugh M.P. (1998). Macroscopic and microscopic properties of a cloned glutamate transporter/chloride channel. J. Neurosci..

[35] Guizy M., David M., Arias C., Zhang L., Cofán M., Ruiz-Gutiérrez V. (2008). Modulation of the atrial specific Kv1.5 channel by the n-3 polyunsaturated fatty acid, alpha-linolenic acid. J. Mol. Cell. Cardiol..

[36] Sherman W., Beard H.S., Farid R. (2006). Use of an induced fit receptor structure in virtual screening. Chem. Biol. Drug Des..

[37] Sherman W., Day T., Jacobson M.P., Friesner R.A., Farid R. (2006). Novel procedure for modeling ligand/receptor induced fit effects. J. Med. Chem..

[38] Friesner R.A., Banks J.L., Murphy R.B., Halgren T.A., Klicic J.J., Mainz D.T. (2004). Glide: a new approach for rapid, accurate docking and scoring. 1. Method and assessment of docking accuracy. J. Med. Chem..

[39] Canul-Tec J.C., Assal R., Cirri E., Legrand P., Brier S., Chamot-Rooke J. (2017). Structure and allosteric inhibition of excitatory amino acid transporter 1. Nature.

[40] Yernool D., Boudker O., Jin Y., Gouaux E. (2004). Structure of a glutamate transporter homologue from Pyrococcus horikoshii. Nature.

[41] Boudker O., Ryan R.M., Yernool D., Shimamoto K., Gouaux E. (2007). Coupling substrate and ion binding to extracellular gate of a sodium-dependent aspartate transporter. Nature.

[42] Verdon G., Boudker O. (2012). Crystal structure of an asymmetric trimer of a bacterial glutamate transporter homolog. Nat. Struct. Mol. Biol..

[43] Akyuz N., Georgieva E.R., Zhou Z., Stolzenberg S., Cuendet M.A., Khelashvili G. (2015). Transport domain unlocking sets the uptake rate of an aspartate transporter. Nature.

[44] Reyes N., Ginter C., Boudker O. (2009). Transport mechanism of a bacterial homologue of glutamate transporters. Nature.

[45] Yazdi S., Stein M., Elinder F., Andersson M., Lindahl E. (2016). The molecular basis of polyunsaturated fatty acid interactions with the Shaker voltage-gated potassium channel. PLoS Comput. Biol..

[46] Feller S.E., Gawrisch K., MacKerell A.D. (2002). Polyunsaturated fatty acids in lipid bilayers: Intrinsic and environmental contributions to their unique physical properties. J. Am. Chem. Soc..

[47] Eldho N.V., Feller S.E., Tristram-Nagle S., Polozov I.V., Gawrisch K. (2003). Polyunsaturated docosahexaenoic vs docosapentaenoic acid-differences in lipid matrix properties from the loss of one double bond. J. Am. Chem. Soc..

[48] Tanui R., Tao Z., Silverstein N., Kanner B., Grewer C. (2016). Electrogenic steps associated with substrate binding to the neuronal glutamate transporter EAAC1. J. Biol. Chem..

[49] Grewer C., Rauen T. (2005). Electrogenic glutamate transporters in the CNS: molecular mechanism, pre-steady-state kinetics, and their impact on synaptic signaling. J. Membr. Biol..

[50] Grewer C., Watzke N., Wiessner M., Rauen T. (2000). Glutamate translocation of the neuronal glutamate transporter EAAC1 occurs within milliseconds. Proc. Natl. Acad. Sci. U. S. A..

[51] Wang J., Albers T., Grewer C. (2018). Energy landscape of the substrate translocation equilibrium of plasma-membrane glutamate transporters. J. Phys. Chem. B.

[52] Akyuz N., Altman R.B., Blanchard S.C., Boudker O. (2013). Transport dynamics in a glutamate transporter homologue. Nature.

[53] Rong X., Zomot E., Zhang X., Qu S. (2014). Investigating substrate-induced motion between the scaffold and transport domains in the glutamate transporter EAAT1. Mol. Pharmacol..

[54] Kortzak D., Alleva C., Weyand I., Ewers D., Zimmermann M.I., Franzen A. (2019). Allosteric gate modulation confers K(+) coupling in glutamate transporters. EMBO J.

[55] Kanner B.I., Bendahan A. (1982). Binding order of substrates to the sodium and potassium ion coupled L-glutamic acid transporter from rat brain. Biochemistry.

[56] Heckman K.L., Pease L.R. (2007). Gene splicing and mutagenesis by PCR-driven overlap extension. Nat. Protoc..

[57] McKhann G.M., 2nd D'Ambrosio R., Janigro D. (1997). Heterogeneity of astrocyte resting membrane potentials and intercellular coupling revealed by whole-cell and gramicidin-perforated patch recordings from cultured neocortical and hippocampal slice astrocytes. J. Neurosci..

[58] Laskowski R.A., W. M M., Moss D.S., Thornton J.M. (1993). The PROCHECK suite of programs provides a detailed check on the stereochemistry of a protein structure. J. Appl. Crystallogr..

[59] Laskowski R.A., Rullmannn J.A., MacArthur M.W., Kaptein R., Thornton J.M. (1996). AQUA and PROCHECK-NMR: Programs for checking the quality of protein structures solved by NMR. J. Biomol. NMR.

