# Supplementary material for: Leucine 434 is essential for docosahexaenoic acid–induced augmentation of L-glutamate transporter current
Source: J Biol Chem. 2022 Dec 9;299(1):102793. doi: 10.1016/j.jbc.2022.102793 (PMC9823230; doi:10.1016/j.jbc.2022.102793)
Supplement: Supplemental Figure S1 [file mmc1.pdf]

A

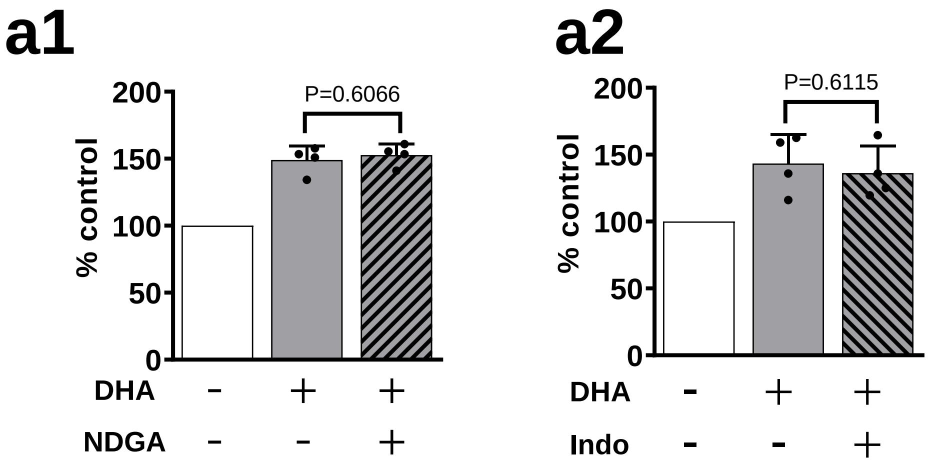

B

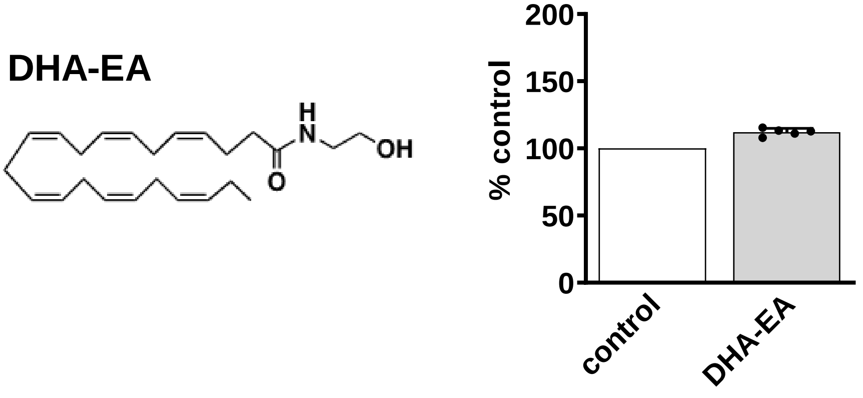

A a1 and a2. DHA metabolites did not affect L-Glu-induced EAAT2 current. Neither nordihydroguaiaretic acid (NDGA, 100 µM), a lipoxygenase inhibitor, nor indomethacin (Indo, 100 µM), a nonselective COX inhibitor, influenced the effect of DHA on L-Glu-induced EAAT2 current.

B. Structures of DHA-ethanolamide (DHA-EA). DHA-EA (100 µM), an uncharged hydrophilic analogue of DHA, had no effect on L-Glu-induced EAAT2 current.

Error bars represent mean ± SD. The numbers written within parentheses in each Figure represent the number of independent experiments. Statistical differences between groups were determined by two-tailed paired Student’s t test. P-values are indicated in each Figure panel.

Fig. S1
