# Supplementary material for: Leucine 434 is essential for docosahexaenoic acid–induced augmentation of L-glutamate transporter current
Source: J Biol Chem. 2022 Dec 9;299(1):102793. doi: 10.1016/j.jbc.2022.102793 (PMC9823230; doi:10.1016/j.jbc.2022.102793)
Supplement: Supplemental Figure S2 [file mmc2.pptx]

## Slide 1
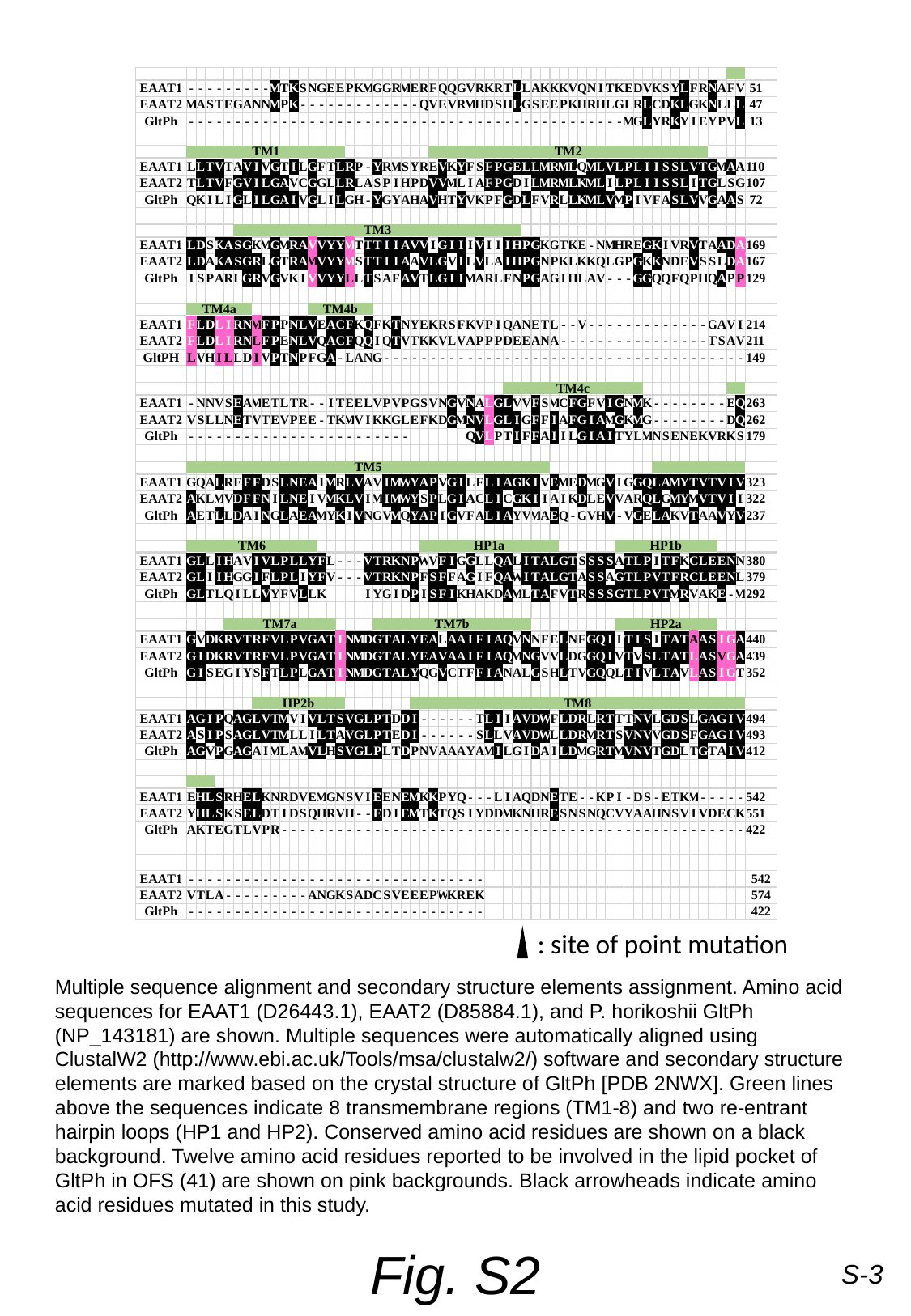

: site of point mutation
Multiple sequence alignment and secondary structure elements assignment. Amino acid sequences for EAAT1 (D26443.1), EAAT2 (D85884.1), and P. horikoshii GltPh (NP_143181) are shown. Multiple sequences were automatically aligned using ClustalW2 (http://www.ebi.ac.uk/Tools/msa/clustalw2/) software and secondary structure elements are marked based on the crystal structure of GltPh [PDB 2NWX]. Green lines above the sequences indicate 8 transmembrane regions (TM1-8) and two re-entrant hairpin loops (HP1 and HP2). Conserved amino acid residues are shown on a black background. Twelve amino acid residues reported to be involved in the lipid pocket of GltPh in OFS (41) are shown on pink backgrounds. Black arrowheads indicate amino acid residues mutated in this study.
Fig. S2
S-3
