# Supplementary material for: Leucine 434 is essential for docosahexaenoic acid–induced augmentation of L-glutamate transporter current
Source: J Biol Chem. 2022 Dec 9;299(1):102793. doi: 10.1016/j.jbc.2022.102793 (PMC9823230; doi:10.1016/j.jbc.2022.102793)
Supplement: Supplemental Figure S3 [file mmc3.pdf]

A

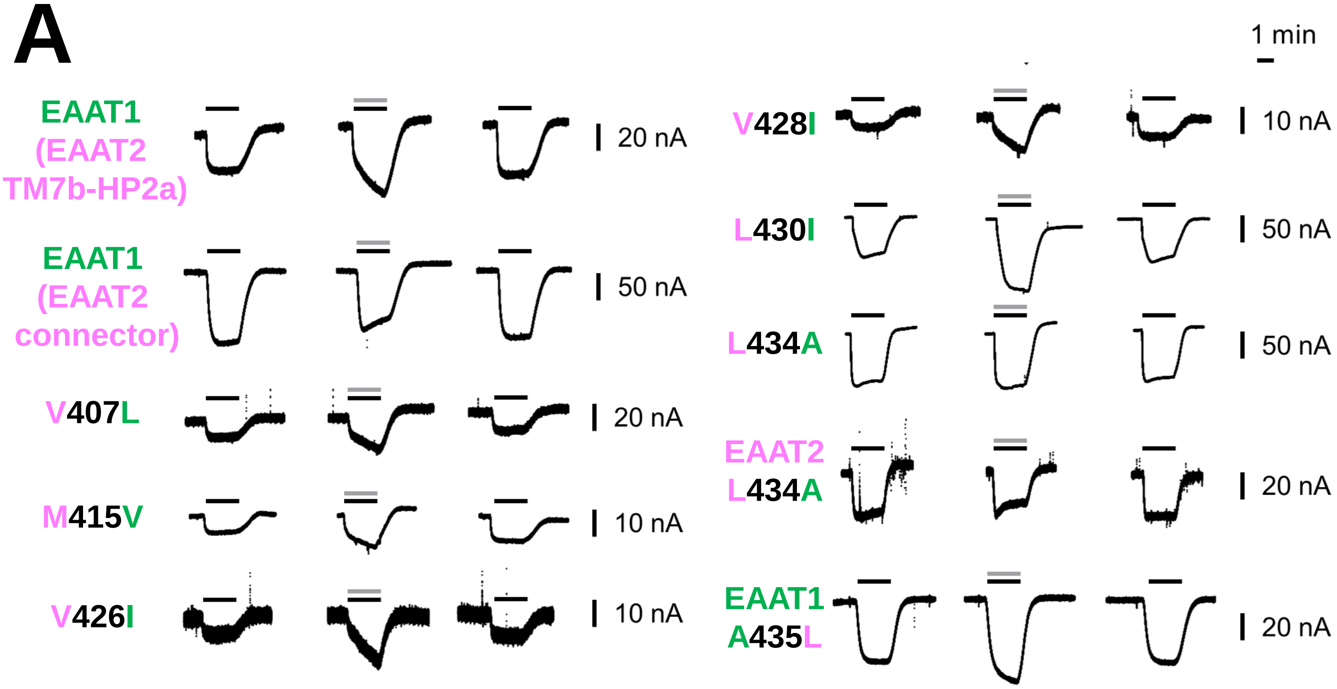

B

Transporter	Mean Absolute value of amplitude (nA)	n
EAAT2	31.4 ± 21.7	27
EAAT1	103.1 ± 17.5	18
EAAT1 (EAAT2 TM7b-HP2a)	25.7 ± 16.3	20
EAAT1 (EAAT2 connector)	94.4 ± 45.7	16
EAAT1 (EAAT2 TM7b-HP2a) V407L	17.7 ± 9.5	14
EAAT1 (EAAT2 TM7b-HP2a) M415V	12.1 ± 4.8	14
EAAT1 (EAAT2 TM7b-HP2a) V426I	10.0 ± 3.4	10
EAAT1 (EAAT2 TM7b-HP2a) V428I	13.4 ± 5.6	9
EAAT1 (EAAT2 TM7b-HP2a) L430I	37.9 ± 28.3	14
EAAT1 (EAAT2 TM7b-HP2a) L434A	52.7 ± 21.2	10
EAAT2 L434A	23.6 ± 10.3	10
EAAT1 A435L	58.0 ± 40.6	14

A. Representative traces for all chimeras and single mutants. The EAATs current induced by L-Glu (50 µM, black bars) in the absence or presence of DHA (100 µM, grey bars) at holding potential of – 50 mV.
B. The absolute value of the EAATs current amplitude induced by L-Glu (50 µM) at – 50 mV.
Error bars represent mean ± SD.

Fig. S3
