# Supplementary material for: Leucine 434 is essential for docosahexaenoic acid–induced augmentation of L-glutamate transporter current
Source: J Biol Chem. 2022 Dec 9;299(1):102793. doi: 10.1016/j.jbc.2022.102793 (PMC9823230; doi:10.1016/j.jbc.2022.102793)
Supplement: Supplemental Figure S4 [file mmc4.pdf]

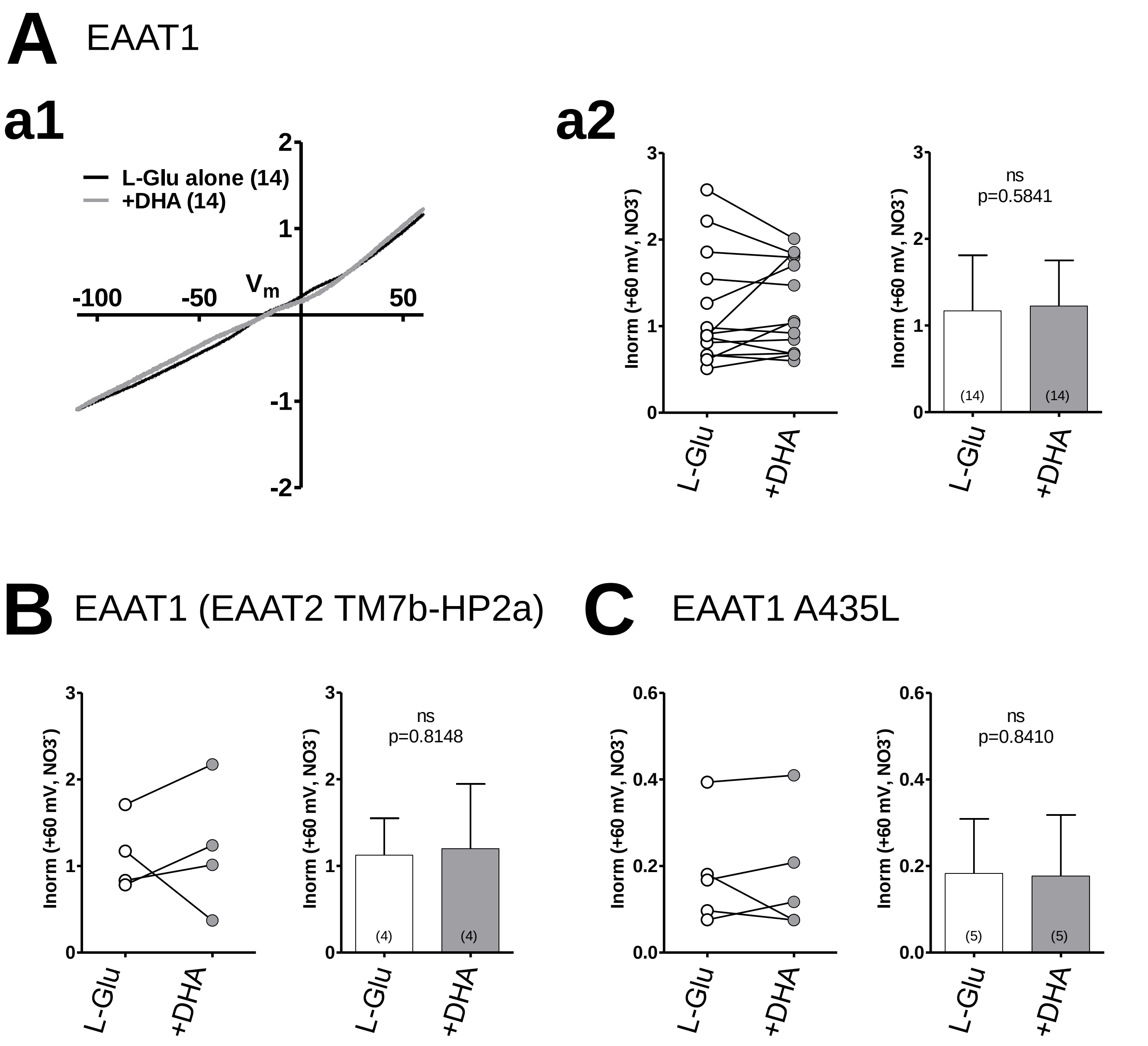

A a1. Current-voltage relationships for L-Glu (50 µM)-induced EAAT1 current in the absence (black) or presence (grey) of DHA (100 µM) in 96 mM NO^{3-}-based ND96 buffer. Data are shown as the values normalized to that obtained with 50 µM L-Glu alone at –100 mV.
a2. Comparison of EAAT1 currents amplitudes at +60 mV in the absence (white) or presence (grey) of DHA (100 µM) in NO^{3-}-based buffer. Left: before and after. Right: mean values.
B Comparison of EAAT1 (EAAT2 TM7b-HP2a) currents amplitudes at +60 mV in the absence (white) or presence (grey) of DHA (100 µM) in NO^{3-}-based buffer. Left: before and after. Right: mean values.
C Comparison of EAAT1 A435L currents amplitudes at +60 mV in the absence (white) or presence (grey) of DHA (100 µM) in NO^{3-}-based buffer. Left: before and after. Right: mean values.
Error bars represent mean ± SD. The numbers written within parentheses in each Figure represent the number of independent experiments. Statistical differences between groups were determined by two-tailed paired Student’s t test. P-values are indicated in each Figure panel.

Fig. S4
