# Supplementary figures and images for: Leucine 434 is essential for docosahexaenoic acid–induced augmentation of L-glutamate transporter current
Source: J Biol Chem. 2022 Dec 9;299(1):102793. doi: 10.1016/j.jbc.2022.102793 (PMC9823230; doi:10.1016/j.jbc.2022.102793)

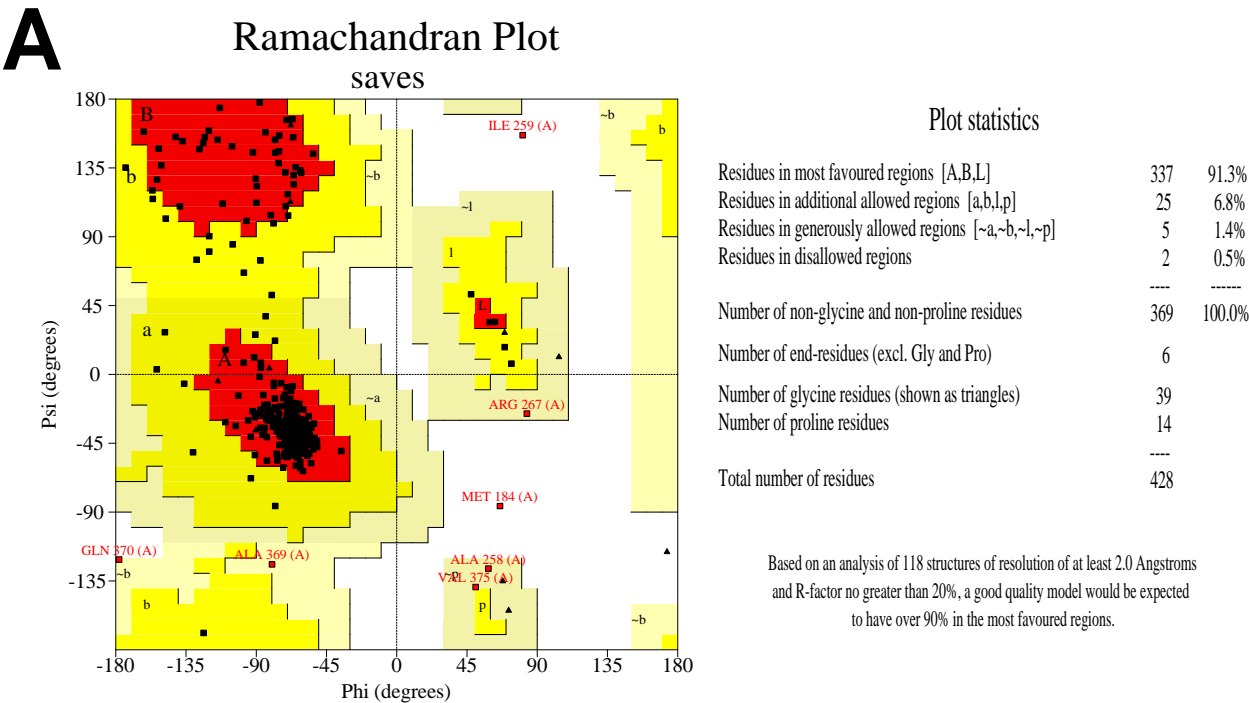

# B

## b1

| Group ID | Entry ID | IFD score          |
|----------|----------|--------------------|
| 1        | 1        | <b>-14861.30 *</b> |
|          | 2        | -14847.69          |
|          | 3        | -14831.39          |
| 2        | 4        | <b>-14850.24 *</b> |
|          | 5        | -14849.27          |
|          | 6        | -14830.42          |
| 3        | 7        | -14826.60          |

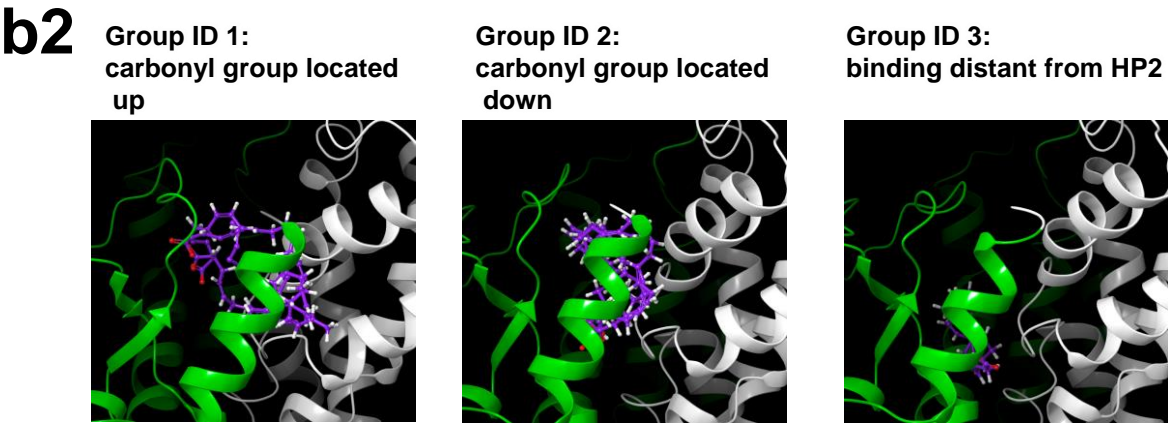

Supplement: Supplemental Figure S5 [file mmc5.pdf]
